# Endogenous TGF-β activation by reactive oxygen species is key to Foxp3 induction in TCR-stimulated and HIV-1-infected human CD4^+^CD25^- ^T cells

**DOI:** 10.1186/1742-4690-4-57

**Published:** 2007-08-09

**Authors:** Shoba Amarnath, Li Dong, Jun Li, Yuntao Wu, WanJun Chen

**Affiliations:** 1 Mucosal Immunology Unit, OIIB, NIDCR, NIH, Bethesda, MD 20895, USA; 2 National Center for Biodefense and Infectious Diseases, Department of Molecular and Microbiology, George Mason University, Manassas, VA 20110, USA

## Abstract

**Background:**

CD4^+^CD25^+ ^T regulatory cells (Tregs) play an important role in regulating immune responses, and in influencing human immune diseases such as HIV infection. It has been shown that human CD4^+^CD25^+ ^Tregs can be induced in vitro by TCR stimulation of CD4^+^CD25^- ^T cells. However, the mechanism remains elusive, and intriguingly, similar treatment of murine CD4^+^CD25^- ^cells did not induce CD4^+^CD25^+^Foxp3^+ ^Tregs unless exogenous TGF-β was added during stimulation. Thus, we investigated the possible role of TGF-β in the induction of human Tregs by TCR engagement. We also explored the effects of TGF-β on HIV-1 infection mediated induction of human Tregs since recent evidence has suggested that HIV-1 infection may also impact the generation of Tregs in infected patients.

**Results:**

We show here that endogenous TGF-β is key to TCR induction of Foxp3 in human CD4^+^CD25^- ^T cells. These events involve, first, the production of TGF-β by TCR and CD28 stimulation and the activation of latent TGF-β by reactive oxygen species generated from the activated T cells. Biologically active TGF-β then engages in the induction of Foxp3. Neutralization of active TGF-β with anti-TGF-β antibody or elimination of ROS with MnTBAP abrogated Foxp3 expression. HIV-1 infection enhanced Foxp3 expression in activated CD4^+^CD25^- ^T cells; which was also abrogated by blockade of endogenous TGF-β.

**Conclusion:**

Several conclusions can be drawn from this work: (1) TCR and CD28-induced Foxp3 expression is a late event following TCR stimulation; (2) TGF-β serves as a link in Foxp3 induction in human CD4^+^CD25^- ^T cells following TCR stimulation, which induces not only latent, but also active TGF-β; (3) the activation of TGF-β requires reactive oxygen species; (4) HIV infection results in an increase in Foxp3 expression in TCR-activated CD25^- ^T cells, which is also associated with TGF-β. Taken together, our findings reinforce a definitive role of TGF-β not only in the generation of Tregs with respect to normal immune responses, but also is critical in immune diseases such as HIV-1 infection.

## Background

CD4^+^CD25^+ ^T regulatory cells (Tregs) have been recognized as the most important immune regulatory cells; they are involved in immune tolerance, autoimmunity, inflammation, transplantation, cancer and HIV infection [1-5]. Human CD4^+^CD25^+ ^Tregs possess most of the basic features of their counterparts in mice [6,7], including specific expression of Foxp3 and immunosuppression of normal CD4^+ ^responder T cells when co-cultured. Although it is generally believed that "natural" CD4^+^CD25^+ ^Tregs are generated from the thymus, the detailed pathways by which these Tregs are developed remain elusive [8-11]. In addition, it has been documented that murine CD4^+^CD25^+ ^Foxp3^+ ^Tregs cannot be generated from peripheral CD4^+^CD25^- ^naive T cells by TCR plus CD28 co-stimulation [10-14] unless exogenous TGF-β is included in the cultures [10,12,15]. In contrast, in humans, some studies have indicated that stimulation of human peripheral CD4^+^CD25^- ^T cells with anti-TCR and anti-CD28 antibodies can generate CD4^+^CD25^+ ^T regulatory cells that also express Foxp3 and are immunosuppressive [16,17]. These findings, although still controversial [15,18], have raised a critical issue, namely, how to reconcile the observed induction of Foxp3 and Tregs with the established paradigm that the primary goal of T cell activation by TCR and CD28 is to induce T cell proliferation and differentiation to mount specific T cell immunity [19]? Nevertheless, the molecular mechanism underlying TCR-induction of Foxp3 in human T cells is not understood. Since TGF-β has been implicated in the induction of Tregs in murine cells, we set out to investigate whether TGF-β has a role in the unexpected induction of Tregs by TCR stimulation in human T cells.

In the human immune system, Tregs play an important role in regulating immune responses, as well as in controlling immune diseases such as infection by viruses that may impair the immune system. The human immunodeficiency virus (HIV) is one such virus, and HIV infection causes gradual depletion of CD4 T cells in the body. Recent evidence has indicated that CD4^+^CD25^+ ^Tregs may play a role in the pathogenesis of HIV infection [20-23]. The involvement of Tregs in HIV-1 infection appears to be complicated and may depend on the site of viral replication and stages of disease progression. In SIV-infected macaques, Tregs were depleted in the GALT, suggesting a virus-mediated loss of Treg function that may facilitate immune activation and productive viral replication [24]. On the other hand, Tregs may also suppress protective cell-mediated immunity against HIV-1. Depletion of Tregs in infected patients enhances anti-HIV T cell responses [25]. Indeed, it has also been shown that the number of FOXP3+ T cells were significantly increased in lymphoid tissues of infected patients [26]. The mechanism has been attributed to HIV-1-mediated promotion of Treg cell survival [26]. However, the possibility of HIV-1-stimulated conversion of non-Tregs to Tregs was not addressed.

In this report, we define a novel molecular mechanism that links TCR stimulation and Foxp3 expression in human CD4^+^CD25^- ^T cells. Notably, these events first involve the production of TGF-β by TCR and CD28 engagement and the activation of TGF-β by ROS produced from the activated T cells. Biologically active TGF-β then engages in the induction of Foxp3. The TCR-induced Foxp3^+^CD25^+ ^T cells exhibit suppressive activity on TCR-driven T cell proliferation in CD4^+ ^T cells in vitro. We also demonstrate that unexpectedly, HIV infection upregulates Foxp3 expression in TCR-activated CD4^+^CD25^- ^T cells, again through TGF-β production. Surprisingly, addition of exogenous TGF-β inhibits HIV replication in CD4^+^CD25^- ^T cells. Our data demonstrate a novel connection of TGF-β and Tregs in HIV infection of T cells that may have implications in the Treg activity observed in vivo in infected patients.

## Results

### Human CD4^+^CD25^- ^T cells express Foxp3 upon TCR stimulation

**Figure 1:**
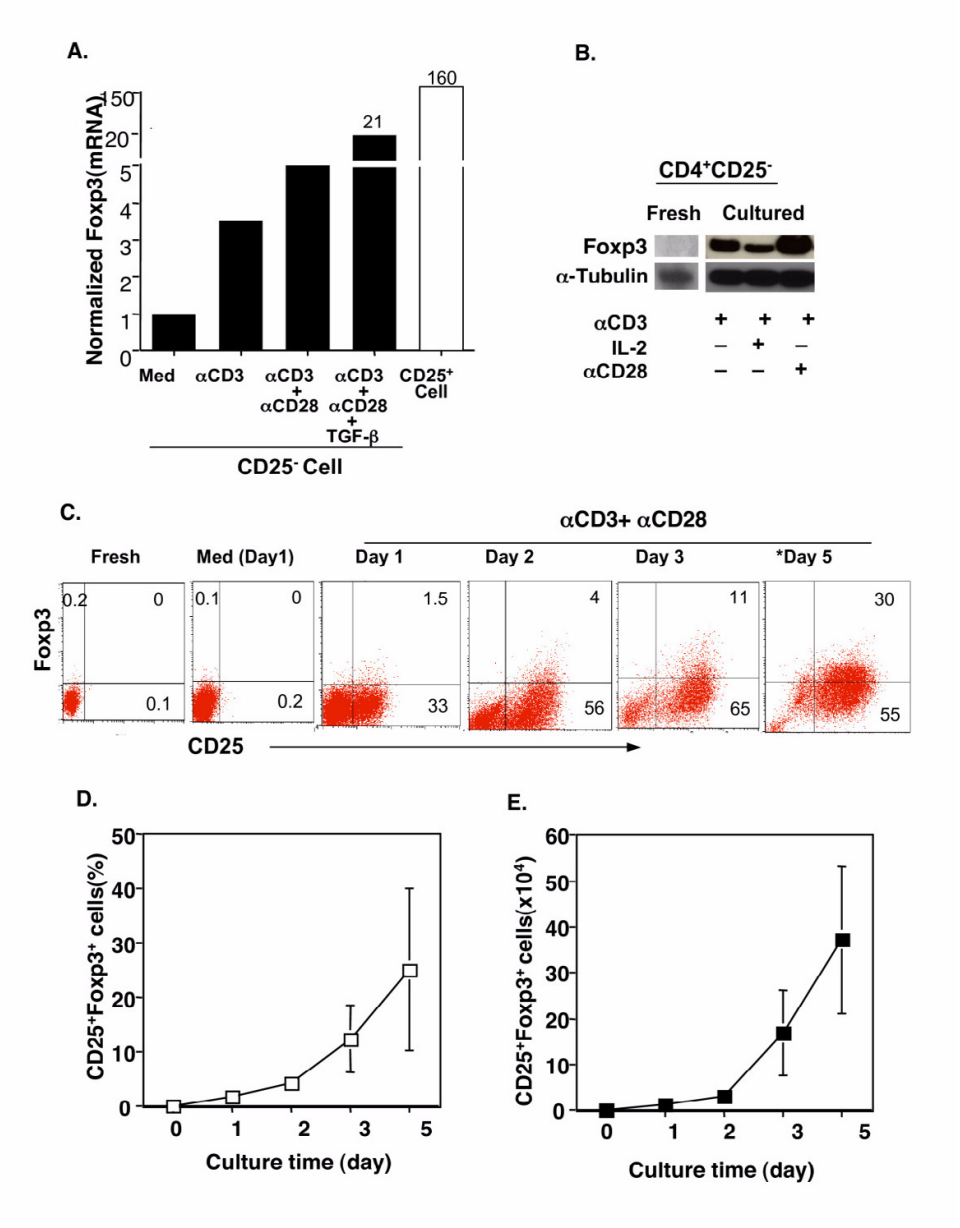
TCR stimulation of human CD4^+^CD25^- ^T cells induces Foxp3. Highly purified CD4^+^CD25^- ^T cells (98–99%) were stimulated with the indicated regimen in X-Vivo 20 serum-free medium, and Foxp3 mRNA and protein were examined. **A**. Cells were cultured for 48–72 hours. RNA was isolated and cDNA synthesized for assessing the expression of Foxp3 by real-time PCR. Freshly isolated CD4+CD25+ T cells were used as a positive control for Foxp3 expression. Values are expressed as the normalized ratio of Foxp3 to GAPDH. **B**. Analysis of Foxp3 protein with Western blot. The experiments were repeated three times with similar results. **C-E**. Analysis of intracellular Foxp3 protein at the single-cell level by FACS. Freshly isolated CD4^+^CD25^- ^cells (Fresh) or cultured cells at the indicated time were stained with FITC-anti-CD25 (surface) and PE-anti-Foxp3 (intracellular) and analyzed on FACS^calibur^. A representative FACS profile is shown as dot plots of CD25 versus Foxp3 (C). The quadrant gates were set according to the negative isotype control antibodies in the respective cells. The kinetics of the percentage (D) and total number (E) of CD25^+^Foxp3^+^cells are shown as Mean ± SD of each group at each time point (n = 3 to 6). Med: Medium; αCD3: anti-CD3 mAb; αCD28: anti-CD28 mAb. * indicates a different donor.

**Figure 2:**
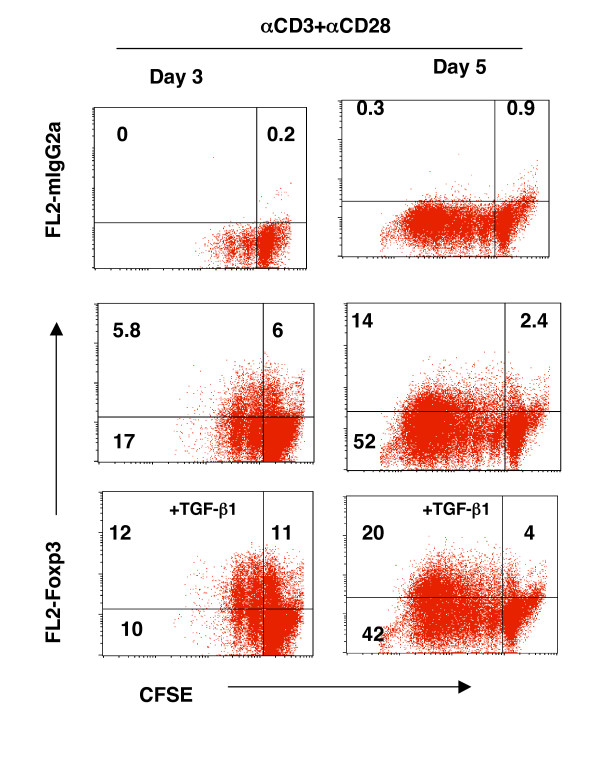
Foxp3^+ ^T cells exist in both non-proliferating (CFSE^+hi^) and dividing (CFSE^+low^) TCR-stimulated CD4^+^CD25^- ^T cells. CD4^+^CD25^- ^T cells were labeled with CFSE (2.5 μM) and cultured with anti-CD3 and anti-CD28 for 3 and 5 days. Cells were then counter-stained intracellularly with PE-conjugated anti-Foxp3 antibody. The cells were analyzed with FACS and a representative profile of CFSE vs. Foxp3 or its control antibody (mIgG2a) is displayed. The experiments were repeated three times with similar results. Data not shown here are the cultures with cells in medium alone. No CFSE dilution (CFSE^+low^) or Foxp3^+ ^cells were observed.

**Figure 3:**
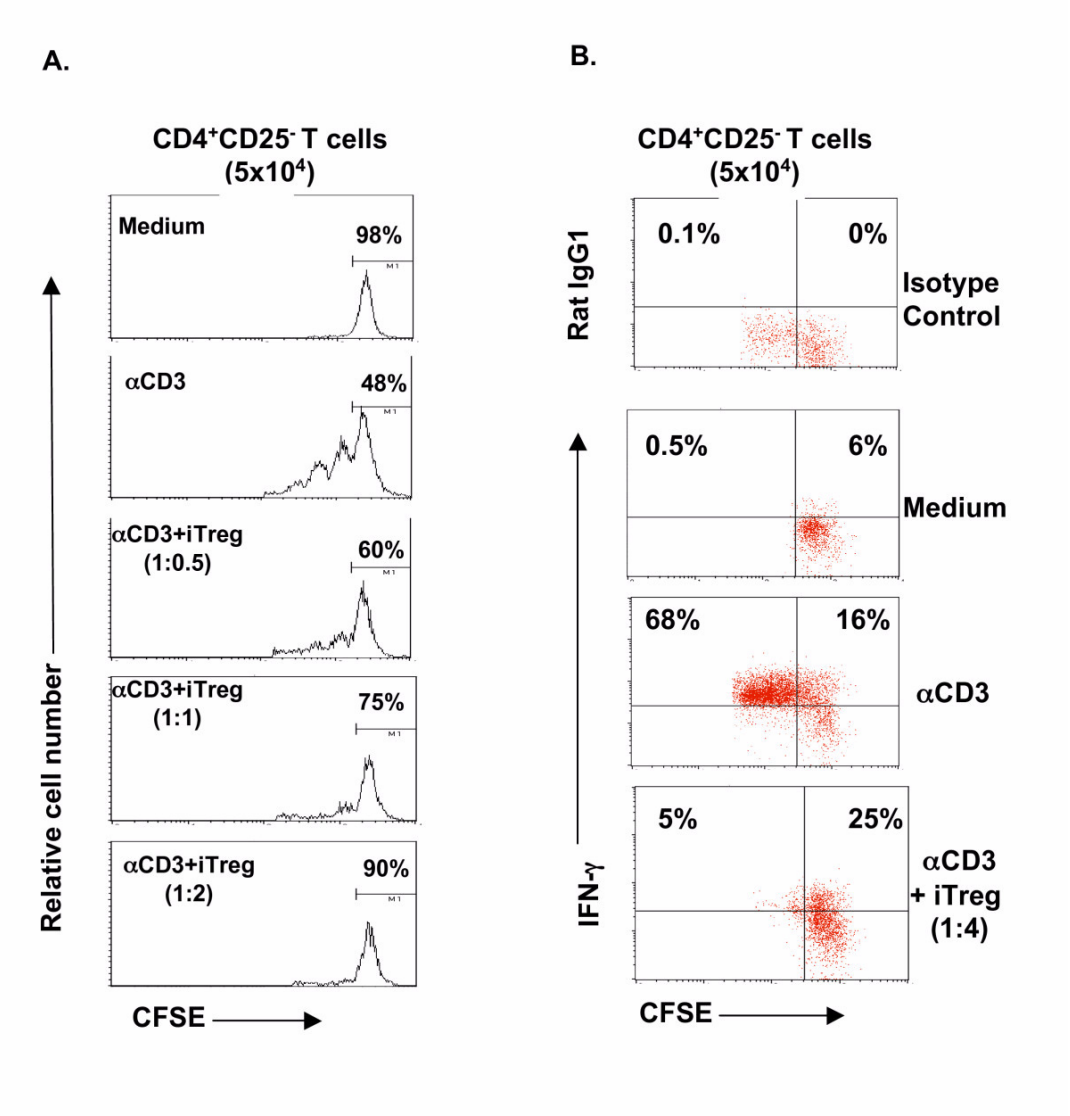
TCR induced CD25^+^Foxp3^+ ^T cells were immunosuppressive to CD4^+^CD25^- ^T cell proliferation in vitro. **A**. CD4^+^CD25^- ^T cells were cultured with anti-CD3 and anti-CD28 for 5 days. The converted CD4^+^CD25^+^Foxp3^+ ^T cells were purified and washed extensively. The converted Tregs were then used at varying concentrations in a co-culture suppression assay along with CD4^+^CD25^- ^(5 × 10^4^) T cells pre-labeled with CFSE as responders and autologous monocytes (2 × 10^5^) as accessory cells. Anti-CD3 antibody was added into the start of the co-culture suppression assay (0.5 μg/ml). CFSE dilution of responder cells was measured after 72 hrs using flow cytometry. **B**. IFN-γ production of responder cells in the co-culture assay as detected by flow cytometry after 72 hrs. iTreg: induced Foxp3^+^CD4^+^CD25^+ ^T cells. The experiment was repeated for three times with similar results.

We first examined whether TCR stimulation of human CD4^+^CD25^- ^T cells induced Foxp3. Human CD4^+^CD25^- ^T cells were purified from peripheral blood of normal healthy donors. As reported [16-18], freshly isolated human CD4^+^CD25^- ^T cells possessed undetectable levels of Foxp3 mRNA and protein (data not shown). TCR stimulation of CD4^+^CD25^- ^T cells with plate-coated anti-CD3 antibody induced detectable Foxp3 mRNA as determined by real-time PCR (Fig. 1A) and protein by Western blot (Fig. 1B) and intracellular Foxp3 staining by flow cytometry (Fig. 1C). Co-stimulation of CD28 further increased Foxp3 expression (Fig. 1A,B,C), whereas exogenous IL-2 did not have an obvious effect (Fig. 1B). Kinetic studies showed that both the percentage (Fig. 1C,D) and total number (Fig. 1E) of CD4^+^CD25^+ ^Foxp3^+ ^T cells were dramatically augmented after 3 days in TCR- and CD28-stimulated CD4^+^CD25^- ^T cell cultures, although they were detectable by days 1 and 2 (Fig. 1C,D,E). As expected and consistent with previous reports [15,27], exogenous TGF-β significantly upregulated Foxp3 expression in TCR-stimulated human CD4^+^CD25^- ^T cells (Fig. 1A, and Fig. 2). Intriguingly, CD25^+^Foxp3^+ ^T cells were found not only in non-divided cells, but also in proliferated cells, when determined by carboxyfluorescein diacetate succinimidyl ester (CFSE) dilution assay and analyzed by flow cytometry (Fig. 2). Of special note. exogenous TGF-β failed to inhibit T cell proliferation under the current optimal (anti-CD3+anti-CD28) culture conditions (Fig. 2). Despite their proliferation, CD25^+^Foxp3^+ ^T cells induced by TCR and CD28 stimulation produced only a trivial amount of intracellular IFN-γ and undetectable IL-4 (data not shown). Similar results were obtained when CD4^+^CD25^-^CD45RO^- ^T cells [18] were stimulated with anti-CD3 and anti-CD28 antibodies (unpublished results). Significantly, when the TCR-induced CD4^+^CD25^+ ^T cells that contained significant number of Foxp3^+ ^T cells (Fig. 1) were co-cultured with autologous CD4^+^CD25^- ^T responder cells in the presence of autologous monocytes as APCs, anti-CD3 driven T cell proliferation was dramatically suppressed (Fig. 3A) and IFN-γ production was inhibited (Fig. 3B), suggesting their biologically regulatory feature. Thus, TCR and CD28 co-stimulation of CD4^+^CD25^- ^T cells induces Foxp3 expression, but the effect is vivid at later stages of cell culture (>2–3 days).

### T cell-derived TGF-β is involved in TCR induction of Foxp3 in human CD4^+^CD25^- ^T cells

**Figure 4:**
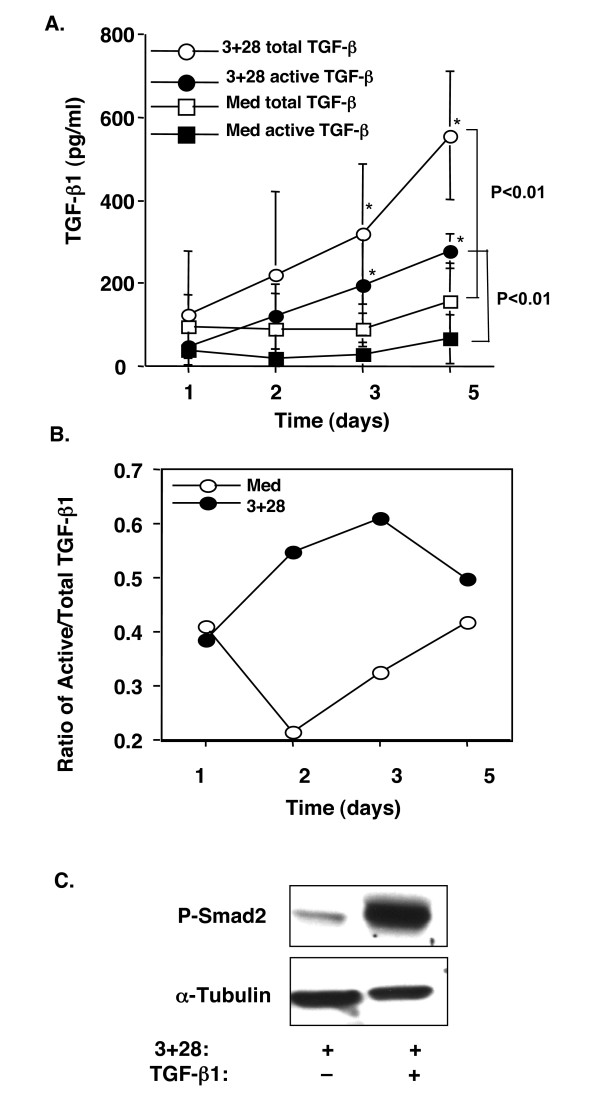
TCR and CD28 stimulation of human CD4^+^CD25^- ^T cells produced TGF-β and exhibited phosphorylation of Smad2. **A**. CD4^+^CD25^- ^T cells (1 × 10^6^/ml) were cultured with anti-CD3 and anti-CD28 in X-Vivo 20 serum-free medium for the indicated time points. Cell-free supernatants were either untreated (for active TGF-β) or treated with 1 N HCl (for total TGF-β) followed by ELISA for TGF-β1 measurement. The values are shown as Mean ± SD of individuals in each group at each time point (n = 3 to 9). **B**. The relative ratio of active to total TGF-β is shown in each time point as in A. **C**. Western blot analysis of P-Smad2 in cultured CD4^+^CD25^- ^T cells (72 hrs). Whole cell lysis protein (70 μg/ml) was loaded into each lane. P-Smad2 was detected with anti-P-Smad2 antibody. α-tubulin was used as host protein control. 3+28: anti-CD3+anti-CD28; Med: medium.

We then sought to determine the underlying molecular mechanism of the Foxp3 expression in TCR-activated human CD4^+^CD25^- ^T cells. We focused on endogenous TGF-β produced by T cells, since previous studies from our own and other independent groups [10,12,15,27,28] have clearly demonstrated that exogenous TGF-β induces Foxp3 expression in mouse and human CD4^+^CD25^- ^T cells (Fig. 1 and Fig. 2). In order to eliminate any possible contamination by exogenous TGF-β contained in the FBS that is usually a component of normal complete culture medium, we used serum-free medium (X-Vivo 20) in our experiments. We first examined whether TCR and CD28 stimulation of human CD4^+^CD25^- ^T cells produced TGF-β. Highly purified human CD4^+^CD25^- ^T cells were cultured with anti-CD3, and the anti-CD28 antibodies and TGF-β in the culture supernatants were measured by ELISA. Since TGF-β is usually secreted as its latent form (LAP-TGF-β), we first studied the total TGF-β protein (the supernatants were acid activated with HCl in vitro). TCR- and CD28-stimulated CD4^+^CD25^- ^T cells secreted TGF-β1 (Fig. 4A). Kinetic studies revealed that TGF-β production was time-dependent (Fig. 4A), with barely detectable levels before 48 hrs, but increased significantly after 72 hrs (Fig. 4A), which was positively correlated with the Foxp3 expression (Fig. 1C,D). Since only biologically active TGF-β (removal of latency-associated peptide [LAP]) can bind to its receptors and execute signal transduction [29], we then measured the levels of active TGF-β (without HCl treatment in vitro) in the cultures. To our surprise, active TGF-β1 was also augmented following the stimulation (Fig. 4A). Importantly, the proportion of active TGF-β to the total TGF-β increased in a time-dependent manner, with about 37% at 24 hrs to almost 65% at 72 hrs (Fig. 4B), whereas the ratio was not changed (even decreased) in medium-treated cultures (Fig. 4B). Finally, TGF-β protein in the CD4^+^CD25^- ^cell lysates was analyzed by western blot. Stimulation of TCR and CD28 induced TGF-β production, which appeared at 48 hrs and further increased thereafter (data not shown). Thus, TGF-β was not only produced and secreted, but also activated by TCR and CD28 stimulation in human CD25^- ^T cells.

**Figure 5:**
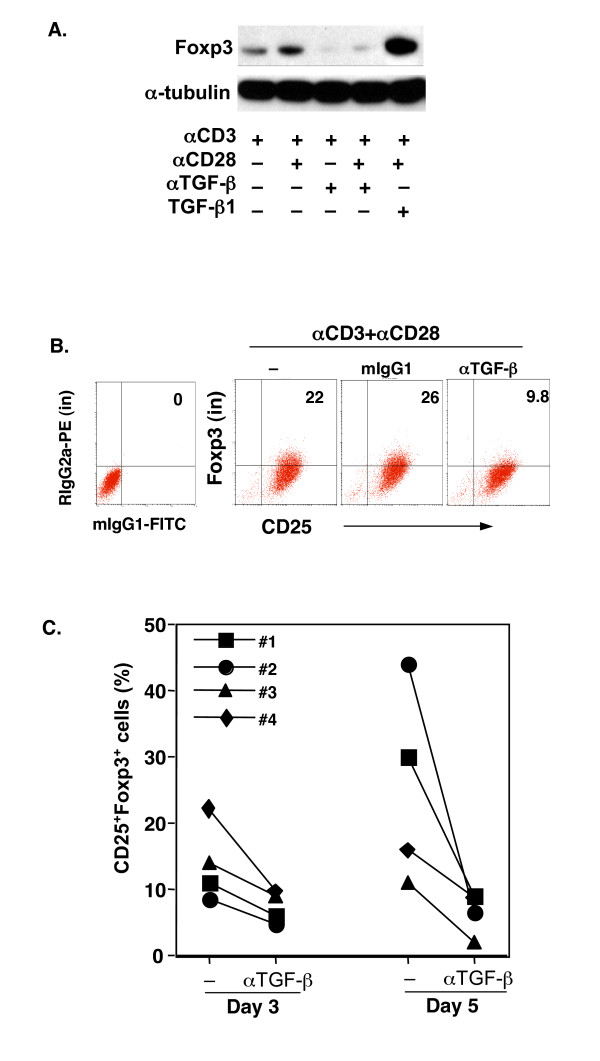
Neutralization of endogenous TGF-β abrogated TCR-induced Foxp3 expression. **A**. Western blot analysis of Foxp3 protein in cultured CD4^+^CD25^- ^T cells with indicated reagents (72 hrs). **B**. FACS analysis of intracellular Foxp3 protein cultured with TCR and CD28 in the presence of anti-TGF-β1,2,3 (αTGF-β) or control (mIgG1) antibodies (72 hr). The data are shown for a representative donor. The values are presented as the percentage of CD25^+^Foxp3^+ ^T cells. **C**. CD25^+^Foxp3^+ ^T cells (%) in the TCR- and CD28-stimulated CD4^+^CD25^- ^T cells in the absence (-) or presence of anti-TGF-β1,2,3 antibody (αTGF-β) at days 3 and 5. Each symbol represents one donor.

To provide evidence that TGF-β signal transduction was activated in TCR- and CD28-stimulated T cells, phosphorylation of Smad2 (P-Smad2), a critical down-stream step in the TGF-β signaling pathway, was examined in TCR-stimulated CD4^+^CD25^- ^T cells. Western blot analysis revealed that P-Smad2 was positive in TCR-stimulated CD25^- ^T cells (Fig. 4C). As a positive control, inclusion of exogenous TGF-β in the cultures dramatically upregulated the levels of P-Smad2 (Fig. 4C), which was positively correlated with the increase in Foxp3^+ ^T cells (Fig. 2). Most importantly, to confirm that the TGF-β produced and activated in TCR-activated CD25^- ^T cells was indeed responsible for Foxp3 expression, an anti-TGF-β monoclonal antibody (clone 1D11) was included in the culture that could abolish all three isoforms of active TGF-β1,2, and 3. Addition of anti-TGF-β antibody dramatically reduced TCR-induced Foxp3 mRNA (data not shown) and protein by either Western blot analysis (Fig. 5A) or by intracellular Foxp3 staining (Fig. 5B,C). Quantitative analysis of the WB bands revealed that neutralization of TGF-β with anti-TGF-β antibodies almost completely abrogated the Foxp3 induction in TCR-stimulated CD4^+^CD25^- ^T cells (more than 200-fold decrease), whereas exogenous TGF-β further enhanced TCR-induced Foxp3 expression (6- and 3.5-fold increase compared to αCD3 and aCD3+αCD28 treated cells respectively). The effect of TGF-β neutralization on Foxp3 reduction was seen most significantly after 72 hrs of culture (Fig. 5C). Despite the great degree of variability among individuals, anti-TGF-β antibody consistently downregulated CD25^+^Foxp3^+ ^T cells (Fig. 5C). Taken together, these data clearly demonstrate that T cell-derived TGF-β is required for TCR induction of Foxp3 in human CD25^- ^T cells in culture.

### TCR and CD28 stimulation produces ROS in human CD4^+^CD25^- ^T cells

**Figure 6:**
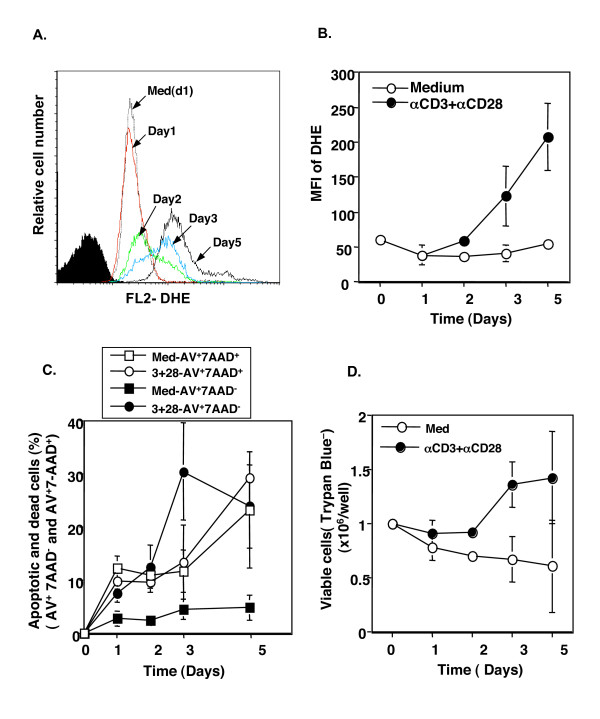
TCR and CD28 stimulation induced ROS production and increased T cell apoptosis. CD4^+^CD25^- ^T cells were stimulated with anti-CD3 and anti-CD28 antibodies for the indicated time points and then intracellular ROS was stained with DHE. The mean fluorescence intensity (MFI) of DHE in a single cell was measured with FACS. A aliquot of cells was stained with Annexin-V and 7-AAD to analyze the early apoptotic (Annexin-V^+^7-AAD^-^) and late apoptotic/dead (Annexin^+^7-AAD^+^) cells. The cells from each cultured well were also examined for viable cells by trypan blue exclusion assay. **A**. A representative histogram profile of DHE staining on the different days. The filled histogram is the un-labeled cells (negative control). **B**. The values are displayed as the Mean ± SD of the MFI of DHE between stimulated (αCD3+αCD28) and non-stimulated (medium) live T cells (R1 gated cells in Fig. S1) at the indicated time points (n = 2 to 5). **C**. The values are presented as the Mean ± SD of the early apoptotic (Annexin^+^7-AAD^-^) and dead/late apoptotic (Annexin^+^7-AAD^+^) between anti-CD3 and anti-CD28 (3+28) and non-stimulated (medium) cells (n = 2 to 5). D. The values are shown as the Mean ± SD of the live cells (trypan blue negative) per well. The original cell number was 1 × 10^6 ^per well(24-well plate; n = 2 to 5).

**Figure 7:**
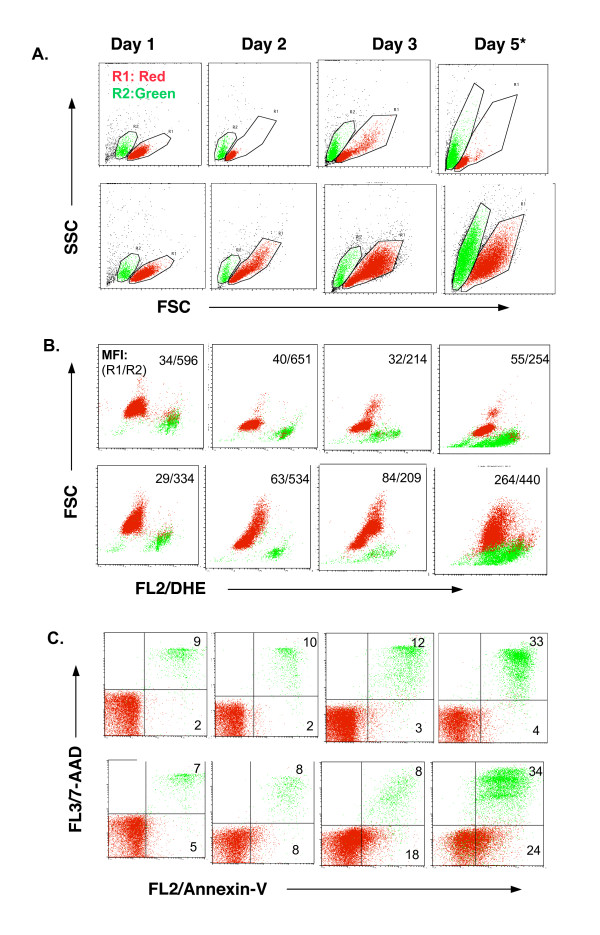
A representative FACS profile of cell size **(A**), DHE fluorescence (**B**) and apoptotic cells (**C**) between anti-CD3 plus anti-CD28-stimulated and medium-treated CD4^+^CD25^- ^T cells is displayed. **A**. Profile of FSC vs SSC is displayed to show the cell size. The cells were electronically gated as two populations based on their size. R1 (red) represents live or early apoptotic cells (see C). R2 (green) represents dead and/or late apoptotic cells (see C). **B**. Profile of DHE fluorescence (ROS^+^) on FL-2 vs. FSC of R1 and R2 cells. The values are shown as the MFI of R1 and R2 cells (R1/R2). Data not shown here are the MFI of unlabeled cells (negative control for DHE staining) on FL2, which is usually < 10. **C**. The profile of Annexin-V vs. 7-AAD staining of cultured cells compensating the R1 and R2 regions as gated in A. The quadrant gates were set according to the negative isotype control antibodies in the respective cells.

Since TCR and CD28 stimulation of human CD4^+^CD25^- ^T cells produced biologically active TGF-β (Fig. 4) that was responsible for Foxp3 induction (Fig. 5), we then studied what caused production of active TGF-β. We focused on ROS that could be produced by TCR stimulation in CD4^+ ^T cells [30-32] and are involved in the activation of latent TGF-β [33-35]. Intracellular ROS can be quantified by staining with dihydroethidium (DHE) that is selectively oxidized by superoxide anion (O_2_^-^) to the fluorescent product ethidium bromide, which can be measured by flow cytometry. Freshly purified CD4^+^CD25^- ^T cells were positive for ROS by DHE staining with low mean fluorescence intensity (MFI = 30–50, Fig. 6B). During the course of cell culture, from days 1 through 5, the MFI of ROS of the live cells in the cultures with medium alone was stable at the baseline level (Fig. 6A, B). Anti-CD3 and anti-CD28 stimulation did not induce any increase in ROS production in the CD25^- ^T cells by day 1 and only slightly enhanced it by day 2 (Fig. 6A,B and Fig. 7). However, by day 3 of cultures, the levels of ROS in TCR- and CD28-stimulated CD25^- ^T cells were dramatically upregulated and continued to increase at day 5 (Fig. 6A,B and Fig. 7B). Interestingly, the increase in ROS in TCR- and CD28-stimulated CD25^- ^T cells was positively correlated with the enhancement of active TGF-β production (Fig. 4A) and Foxp3 expression (Fig. 1). Significantly, anti-CD3 and anti-CD28 stimulation gradually up regulated apoptotic cells (Annexin-V^+^7-AAD^-^) when compared with the medium-alone control cultures (Fig. 6C and Fig. 7C), despite the similar percentages of late apoptotic/dead cells between the two conditions (Fig. 7C). Consequently, the total apoptotic/dead cells (Annexin-V^+^7-AAD^- ^and Annexin-V^+^7-AAD^+^) were elevated dramatically in TCR- and CD28-stimulated cultures after 3 days (Fig. 6C, 7C), which corresponded with the increase in ROS production (Fig. 6C, 3B). Of special note, the dead/late apoptotic T cells were smaller (Fig. 7A, R2 green) and stained positive for Annexin-V and 7-AAD (Fig. 7C). They exhibited higher MFI of ROS (Fig. 7B) than that of live (Annexin-V^-^7-AAD^-^) or early apoptotic (Annexin^+^7-AAD^-^) cells in the same cultures (Fig. 7B). Despite the increase in apoptotic/dead cells, TCR and CD28 co-stimulation enhanced the overall total number of live cells (Trypan blue negative) in the culture, whereas the total number in the medium-alone wells was decreased (Fig. 6D).

**Figure 8:**
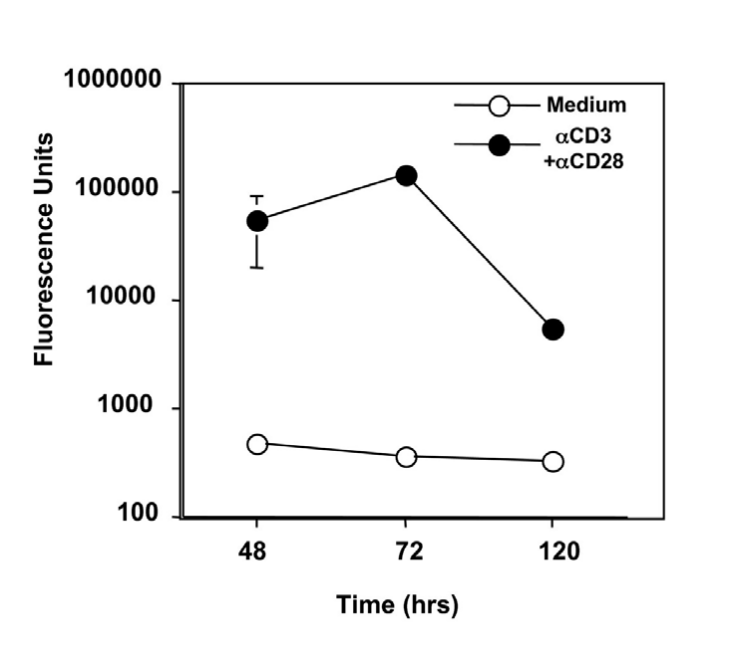
The cell-free supernatant from TCR stimulated CD4^+^CD25^- ^T cell culture contained ROS. CD4^+^CD25^- ^T cells were cultured with anti-CD3 and anti-CD28 for the indicated time points and ROS in the culture supernatant was detected using DCFH-DA as described in the Method section. Oxidation of DCFH-DA was measured using a spectrofluorometer at wavelength 485/535 nm and is represented as fluorescent units. The experiment was repeated twice with similar results.

To determine the presence and amount of extracellular ROS, the supernatants from the activated T cells were incubated with a non-fluorescent 2'7'-dichlorofluorescin-diacetate (DCFH-DA) that could be oxidized into fluorescent 2', 7'-dichlorofluorescein (DCF) by ROS in aqueous solution at 37°C. As expected, the supernatants from the cultures with medium alone had undetectable ROS (Fig. 8) and remained unchanged during days 2,3, and 5 as reflected by a consistent background of DCF fluorescence(Fig. 8). However, the supernatants in the cultures of CD25^- ^T cells stimulated with anti-CD3 and anti-CD28 antibodies contained large amounts of ROS (Fig. 8). The extracellular ROS was significantly enhanced by 48 hours and reached the peak at 72 hours (Fig. 8). Thus, TCR activated CD4^+^CD25^- ^T cells produce ROS and also release them into the cultures.

### ROS produced in activated CD4^+^CD25^- ^T cells are associated with Foxp3 induction through activation of TGF-β

**Figure 9:**
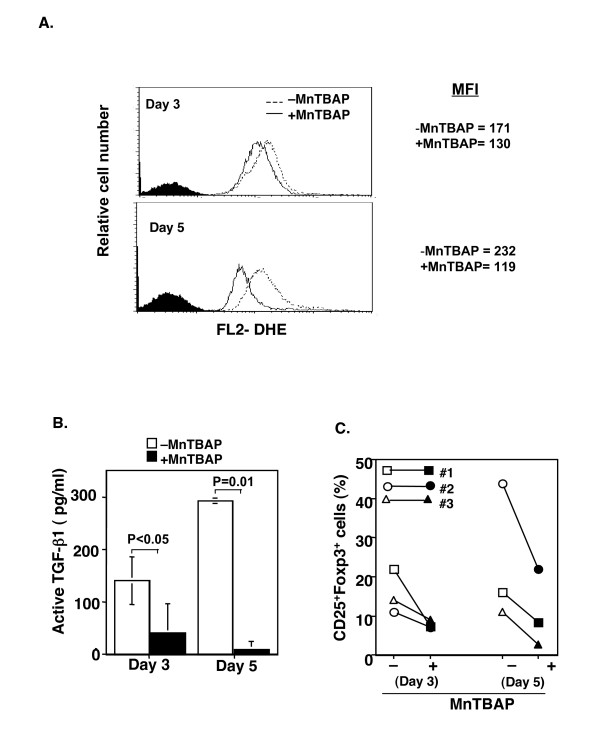
Neutralization of ROS with MnTBAP abrogated active TGF-β and reduced CD25^+^Foxp3^+ ^T cells. CD4^+^CD25^- ^T cells were cultured with anti-CD3 and anti-CD28 antibodies in the presence or absence of MnTBAP (100 μM) for 3 and 5 days. The intracellular ROS production was determined by DHE staining. Active TGF-β in the supernatants was determined with ELISA. The intracellular Foxp3 protein was determined by FACS staining. **A**. A representative overlay of histograms of ROS in the cultured T cells with (+MnTBAP) and without (-MnTBAP) MnTBAP at days 3 and 5. The filled histograms were from unlabeled cells as negative control for DHE staining. The experiment was repeated three times with similar results. **B**. The values are shown as the Mean ± SD of active TGF-β1 in the culture supernatants at days 3 and 5 (n = 2). **C**. MnTBAP reduced CD25^+^Foxp3^+ ^T cells. Each symbol represents one individual.

To determine the role of ROS in the upregulation of Foxp3 expression through activation of TGF-β, a superoxide dismutase mimetic, Mn(III)tetrakis (5,10,15,20-benzoic acid) porphyrin (MnTBAP), that inhibits intracellular and neutralizes extracellular ROS [32,34,36] was included in the CD3 and CD28 co-stimulated CD4^+^CD25^- ^cell cultures. The addition of MnTBAP significantly reduced ROS production in TCR stimulated CD25^- ^T cells; the reduction was most obvious at days 3 and 5 (Fig. 9A,B). Importantly, the active TGF-β was almost completely abrogated in the same cultures with MnTBAP (Fig. 9B), although the total TGF-β was also decreased (data not shown). Unexpectedly, when intracellular Foxp3 was examined, it was found that the TCR-induced CD25^+^Foxp3^+ ^T cells were dramatically reduced in MnTBAP treated cells (Fig. 9C). Thus, ROS produced by TCR activated CD25^- ^T cells plays a role in active TGF-β production, and the TGF-β production in turn induces Foxp3 expression in human CD4^+^CD25^- ^T cells.

### HIV infection upregulates Foxp3 expression in TCR-activated CD4^+^CD25^- ^T cells via TGF-β

**Figure 10:**
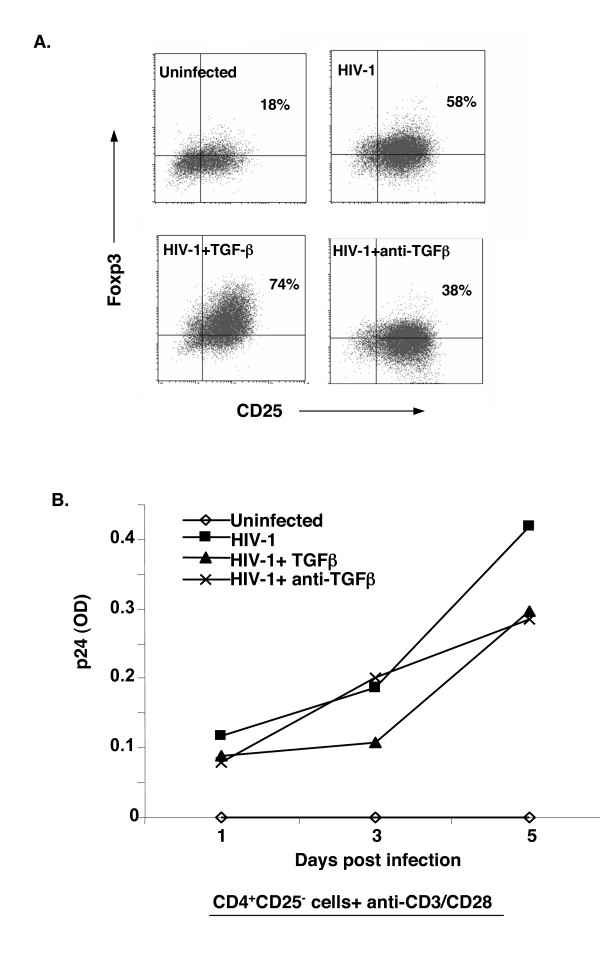
HIV infection upregulated Foxp3 expression in TCR activated CD4^+^CD25^- ^T cells. **A**. Purified CD4^+^CD25^- ^T cells were infected with HIV1 (HIV NLA-3) and cultured with anti-CD3 and anti-CD28 antibodies in the absence (HIV-1) or presence of active TGF-β1 (2 ng/ml) (HIV-1+TGF-β) or anti-TGF-β1,2,3 antibody (HIV-1+anti-TGF-β) for 3 (data not shown) and 5 days. T cells were then stained for surface CD25 and intracellular Foxp3. A representative of two experiments is displayed. A parallel culture of TCR- and CD28-stimulated CD25^- ^T cells without HIV infection (uninfected) was used as control. **B**. The supernatants from the same cultures in A were collected at the indicated time points and tested for HIV p24 with ELISA. The experiment was repeated twice with similar results.

Although CD4^+^CD25^+ ^Tregs have been indicated in the pathogenesis of HIV infection, it is unknown how these Tregs are generated and regulated. We further studied whether HIV infection and replication affected Foxp3 expression in human CD4^+^CD25^- ^T cells. Purified CD4^+^CD25^- ^T cells were infected with HIV for 2 hours, followed by extensive washes to remove any unbound virus [37]. The HIV-infected CD25^- ^T cells were then cultured with anti-CD3 and anti-CD28 antibodies in serum-free X-Vivo medium. Intracellular Foxp3 was examined at days 3 and 5 by flow cytometry. Surprisingly, HIV infection dramatically increased Foxp3 expression in TCR-stimulated CD25^- ^T cells compared to those without virus infection (58% vs. 18%) (Fig. 10A). Consistent with the data of uninfected CD25^- ^T cells (Fig. 5), neutralization of TGF-β with anti-TGF-β1,2,3 antibody reduced Foxp3 expression in HIV-infected CD25^- ^T cells (Fig. 10A), although the antibody had little effects on HIV replication (Fig. 10B). On the other hand, addition of exogenous active TGF-β further enhanced Foxp3 expression in HIV infected cells, which reached about 70–80% of CD25^+^Foxp3^+ ^cells at day 5 (Fig. 10A). Thus, HIV infection enhances Foxp3 expression through TGF-β in TCR-activated human CD25^- ^T cells.

## Discussion

Of the many unresolved questions regarding Tregs, the issue of the factors and/or molecules regulating Foxp3 expression remains significant. In this report, using a serum-free culture system, we have provided the first evidence that endogenous TGF-β produced by TCR and CD28 engagement and activated by ROS plays a critical role in Foxp3 induction in human CD4^+^CD25^- ^T cells. Several important conclusions can be drawn from the current work.

Firstly, TCR and CD28 stimulation indeed upregulates Foxp3 expression at both the mRNA and protein levels, but the significance is only seen later (>2–3 days) in the cultures, suggesting that Foxp3 expression (generation of Tregs) is not an early event during T cell responses. Interestingly, Foxp3^+^CD25^+ ^T cells can be observed not only in non-dividing (CFSE^hi^), but also in proliferating populations (CFSE^low^). However, it remains unknown whether Foxp3 is induced first, followed by T cell proliferation, or induced during or after proliferation. The de novo induction of Foxp3 is supported by the observation that CD4^+^CD25^-^CD45RO^- ^T cells express Foxp3 upon TCR and CD28 stimulation (Fig. 4), although they lack Foxp3 mRNA and protein before culture [18].

Secondly, TGF-β serves as a link in TCR-induction of Foxp3 in human CD4^+^CD25^- ^T cells. TCR and CD28 stimulation of CD25^- ^T cells produces not only latent, but also active TGF-β in the cultures. Intriguingly, active TGF-β is not produced significantly until later in cell culture; this timing correlates with the kinetics of Foxp3 expression in T cells. The levels of remaining active TGF-β in the culture supernatants reach 200–300 pg/ml (per 1 × 10^6 ^cells) after day 3, which is a dose approximately sufficient to induce significant Foxp3 in murine CD4^+^CD25^- ^T cells [12] (data not shown). It should be noted that TCR and CD28 stimulation alone fails to induce detectable levels of active TGF-β and induces minimal total TGF-β in the culture supernatants when murine CD4^+ ^or CD4^+^CD25^- ^T cells (1 × 10^6^/ml) are used [38,39] (our unpublished data). The lack of sufficient TGF-β production may explain the ineffectiveness of Foxp3 induction in murine CD4^+^CD25^- ^T cells upon TCR and CD28 stimulation [10,12,15,27]. The fact that exogenous active TGF-β further upregulates Foxp3 expression in TCR-stimulated CD25^- ^human T cells provides additional evidence of its indispensable role. Of note, under the optimal stimulation (anti-CD3 and anti-CD28), exogenous TGF-β1 (2 ng/ml) fails to inhibit T cell proliferation (Fig. 2), but still dramatically augments Foxp3^+ ^T cells. The failure of TGF-β inhibition of T cell proliferation might be attributed to the high levels of IL-2 induced by optimal signals (CD3+CD28) [40,41]. The data, however, argue against the notion that TGF-β may selectively inhibit CD25^-^Foxp3^- ^T cells, consequently enhancing the frequency of the minor population of CD25^-^Foxp3^+ ^T cells. In support of a critical role of TGF-β signaling in the induction of Foxp3, we have observed that CD4^+^CD25^- ^T cells from conditional knockout mice with T-cell specific deletion of TGF-**β **receptor I fail to become Foxp3^+ ^Tregs upon TCR and exogenous TGF-β stimulation in cultures (unpublished data). Moreover, Smad2, a critical mediator of the TGF-β signal pathway, is phosphorylated in anti-CD3 and CD28 cultured cells, indicating that the TGF-β signal is transduced. Most importantly, depletion of active TGF-β in the cell cultures with anti-TGF-β1,2,3 antibody abrogated TCR and CD28 induced Foxp3 expression; such abrogation is also evident in TCR-activated CD4^+^CD25^-^CD45RO^- ^T cells (data not shown). Thus, TGF-β is a key molecule in TCR-induction of Foxp3 in human CD4^+^CD25^- ^T cells.

Now that endogenous TGF-β has been established to be responsible for TCR induction of Foxp3 in human CD4^+^CD25^- ^T cells, one immediate question is how biologically active TGF-β is produced. TGF-β is usually secreted as a latent form in which the active portion is masked with a latency-associated peptide (LAP) [42]. Newly produced latent TGF-β has to be activated by removing the LAP in order to bind to its receptors and execute signal transduction [43]. Since both latent and active TGF-β are present in the supernatants of TCR- and CD28-stimulated cultures, and indeed active TGF-β is the functional molecule, the question remains as to how latent TGF-β is activated.

The finding that TGF-β is produced/secreted at the late stage of T cell activation, following maximal secretion of most Th1 and Th2 cytokines, may resolve the current paradox of TCR and CD28 induction of Foxp3 (a regulatory T cell gene) and consequent immune tolerance. It is possible that timing is a critical factor. The primary goal of a T cell response by TCR and CD28 stimulation is to produce IL-2 that enables T cells to proliferate and differentiate into Th1 and/or Th2 cells to mount specific immunity [19]. Afterwards, a suppressive factor, TGF-β, is produced/activated, which would provide a "brake" to control/prevent unwanted T cell responses through a negative feedback mechanism that includes induction of Foxp3 and regulatory T cells. As a critical step in this process, the ROS produced by TCR-activated T cells plays a significant role in converting the latent TGF-β into the biologically active form, as neutralization/inhibition of ROS with MnTBAP almost completely abrogates the active TGF-β in the supernatants and significantly decreases the Foxp3 expression in activated CD4^+^CD25^- ^T cells. TCR stimulation increases the level of ROS in T cells [30,36]. The data that the culture supernatants in TCR stimulated CD25^- ^T cells contain large amounts of ROS have provided further evidence that ROS plays a significant role in activation of endogenous TGF-β produced by T cells, although other factors may also participate in this conversion. However, it remains largely unknown where and how ROS is produced in a T cell, and when and how ROS is released from the cell. The mitochondria appears to be one of the largest sources of ROS within cells [34,36,44]. Resting T cells produce low levels of ROS. TCR activation likely triggers increased respiratory activity from the mitochondria to meet the energy requirement for acquisition of effector cell functions [36]. It is of note that the increase in ROS production in T cells is concurrent with increased mitochondrial membrane hyperpolarization, which may lead to ROS re-distribution into the cytosol to initiate cell death. The late apoptotic and/or dead cells may release ROS into the cultures to activate latent TGF-β [34,35]. Indeed, the apoptotic T cells in the TCR- and CD28-treated CD4^+^CD25^- ^T cell cultures accumulate dramatically after 72 hrs, and these late apoptotic/dead T cells (Annexin-V^+^7-AAD^+^) express much higher levels of intracellular ROS than viable and early apoptotic T cells. Although it is unclear how MnTBAP antagonizes ROS, it is conceivable that MnTBAP may reduce intracellular ROS production and abolish the released ROS [32,34,36].

The unexpected finding that HIV infection results in an increase in Foxp3 expression in TCR activated CD25^- ^T cells compared to those without HIV infection uncovers a link between HIV infection and induction of Foxp3 and T regulatory cells. The Foxp3 upregulation was again produced through TGF-β in the T cells, since deletion of TGF-β with anti-TGF-β antibody abrogates the effect. Intriguingly, despite the reduction in Foxp3^+ ^T cells achieved by inhibiting endogenous TGF-β induced by HIV, the replication of the virus was not significantly affected. However, exogenous TGF-β always inhibits HIV replication in activated CD4^+^CD25^- ^T cells in different experimental regimens (Fig. 10B). These data suggest that the dose and the time are the determining factors for TGF-β in regulating HIV. Early and sufficient amounts of active TGF-β (e.g exogenous active TGF-β) present during the initiation of T cell activation may be required for this effect, whereas the TGF-β produced following T cell activation/proliferation might be too late to affect HIV replication in the same cells (e.g. anti-TGF-β inclusion). Consistent to this notion, it has been reported that SIVmac infection in macaques (MACs) is unable to respond to TGF-β1, whereas the non-pathogenic SIVagm infection in African green Monkeys(AGMs) exhibits more sensitivity to TGF-β signaling characterized by longer lasting upregulation of Smad4 [45]. However, the T cell derived TGF-β and induced Foxp3^+ ^T regulatory cells may subsequently inhibit the other neighboring CD4^+ ^T cells exposed to HIV. This notion is supported by the observation that HIV replication in activated natural CD4^+^CD25^+ ^human T cells (a mixture of CD25^+^Foxp3^+ ^regulatory cells and CD25^+^Foxp3^-^non-regulatory T cells) was significantly delayed compared to that in the parallel CD25^- ^T cells (unpublished data). Alternatively, TGF-β inhibition of HIV replication might not be directly associated with Foxp3 expression, thereby suggesting that Foxp3^+ ^T cells are equally susceptible to HIV replication [23]. However, this possibility remains to be investigated. Nevertheless, our data shed new light on the pathogenesis of HIV infection by linking the virus with the induction of Foxp3^+ ^T cells through TGF-β, thereby opening a new avenue to help develop new targets to block HIV infection and replication.

## Methods

### Antibodies and reagents

The human T regulatory cell isolation kit and PE anti-human CD25 antibody were obtained from Miltenyi Biotech (Auburn, CA). The PE-conjugated Foxp3 antibody (clone PCH101) for flow cytometric staining and the rat serum anti-Foxp3 polyclonal antibody for Western blotting were obtained from eBioscience (San Diego, CA). The following antibodies were purchased from BD Bioscience (San Diego, CA): purified mouse anti-human CD3 (clone UCHT1; NA/LE™), purified mouse anti-human CD28 (clone CD28.2; NA/LE™), FITC anti-human CD25 (clone M-A251), FITC anti-human CD4 (clone RPA-T4), PE anti-human CD45RO (clone UCHL1), APC anti-human IL-4 (clone MP4-25D2), APC anti-human IFN-γ (clone B27), and the corresponding isotype-matched negative controls. Anti-phosphorylated Smad2 antibody was from Cell Signaling (Danvers, MA); the biotinylated anti-goat and anti-rabbit antibodies were obtained from Santa Cruz (Santa Cruz, CA). Recombinant TGF-β1, anti-TGF-β1,2,3 and mouse IgG1 isotype control were purchased from R&D systems (Minneapolis, MN). Dihydroethidium was purchased from Molecular Probes. Manganese (III) tetrakis (5,10,15,20-benzoic acid) porphyrin (MnTBAP) was procured from Calbiochem (La Jolla, CA). For detecting ROS, 2'7'-dichlorofluorescin-di-acetate (DCFH-DA) was purchased from Molecular probes (CA, USA). 2',7'-dichlorofluorescein (DCF) was obtained from (Sigma, MO). DCFH-DA was dissolved in ethanol at a concentration of 10 mg/ml. Stock solutions of DCF were also prepared in ethanol.

### Isolation of subsets of human CD4^+ ^T cells

Human peripheral blood mononuclear cells (PBMC) were obtained by leukopheresis of normal volunteers at the Department of Transfusion Medicine at the National Institutes of Health (NIH, Bethesda, MD). The CD4^+^CD25^- ^T cells were isolated using the human T regulatory isolation kit from Miltenyi Biotec following the manufacturer's recommendations. The CD4^+^CD25^- ^T cell population obtained was used for all the assays. Purity of the cells obtained using this method was 98–99% as determined by flow cytometric screening. For further separation of the CD25^- ^population, CD45RO beads (Miltenyi) were used to isolate the CD4^+^CD25^-^CD45RO^+ ^and CD4^+^CD25^-^CD45RO^- ^T cells. In some experiments, CD4^+^CD25^- ^CD45RO^- ^or CD45RO^+ ^T cells were isolated using a Moflo cell sorter (Dako, Colorado).

### Cell culture

Purified CD4^+^CD25^- ^T cells were cultured in serum-free culture medium X-Vivo 20 (BioWhittaker, Walkersville, MD) in 24-well flat-bottom plates. Plates were coated with mouse anti-human CD3 antibody (5 μg/ml in PBS) for 3 hrs at 37°C and washed twice with sterile PBS. Soluble mouse-anti-human CD28 (1 μg/ml) was added to all conditions, recombinant TGF-β1 was added (2 ng/ml) as one experimental condition, the anti-TGF-β1,2,3 antibody (20 μg/ml) was added as another, and the isotype-matched negative control (mIgG1, 20 μg/ml) in some other instances. Another experimental condition was set up in which MnTBAP (100 μM) was added to the culture in addition to anti-CD3 and anti-CD28 stimulation. With respect to assays involving CFSE, freshly isolated CD4^+^CD25^- ^T cells were incubated with 2.5 μM of CFSE in PBS at 37°C for 10 mins, washed twice with X-Vivo 20 medium (BioWhittaker), and then used in culture.

### Real-time PCR for Foxp3 expression

The cell cultures were harvested after 48 hr and 72 hr, and real time PCR was performed as described previously(18) using the cDNA as template for Foxp3 expression and glyceraldehydes-3 phosphate dehydrogenase (GAPDH) was used as the internal control. The Foxp3-specific primers used were 5'-CAG-CAC-ATT-CCC-AGA-GTT-CCT-C-3' and 5'-GCG-TGT-GAA-CCA-GTG-GTA-GAT-C-3', and the Foxp3 probe was 5'-FAM-TCC-AGA-GAA-GCA-GCG-GAC-ACT-CAA-TG-TAMRA. Pre-developed Taqman assay reagent for human GAPDH was used as the internal control. Normalized values for Foxp3 mRNA expression in each sample were calculated as the relative quantity of Foxp3 divided by the relative quantity of GAPDH.

### Western blot analysis

Cells were lysed using cell lysis buffer obtained from Sigma Aldrich, USA. Western blotting was performed as previously described [12] with antibodies to Foxp3 (rat polyclonal IgG, 1:1200), smad2/3 (goat polyclonal IgG, 1:1000), P-Smad2 (rabbit polyclonal IgG, 1:700), TGF-β1(rabbit polyclonal IgG, 1:1000, Promega), α-tubulin (mouse polyclonal IgG, 1:2000) and actin (goat polyclonal IgG, 1:1000, Santa Cruz), followed by horseradish peroxidase-conjugated goat anti-rat IgG, goat anti-mouse IgG or goat anti-rabbit IgG as recommended by the manufacturer.

### Cytokine determination

For cytokine detection, supernatants were collected from the T cell cultures. Total and active TGF-β1 present in the supernatant were determined by ELISA (Promega. WI) as described previously [34]. A standard curve was generated using the known amounts of the purified recombinant TGF-β1 that are provided with the kit.

### Flow cytometric staining

The cells were re-suspended in PBS containing 1% BSA and 0.1% sodium azide. For the staining of cell surface markers, cells were incubated with FITC or PE antibodies for 30 mins at 4°C. Isotype-matched negative controls were used for all flow cytometric analysis. For intracellular IL-2, IL-4, IFN-γ, and Foxp3 staining, the protocol provided with the Foxp3 antibody kit was followed (eBioscience, CA).

### Detection of ROS in T cells

The reactive oxygen species (ROS) in T cells was detected as previously described(Chen et al, 2001). The cells were resuspended in 1 ml of colorless DMEM containing 2.5 μM dihydroethidium (DHE). The cells were incubated at 37°C for 40 min, washed once with the medium, resuspended in PBS containing 1% BSA, and then subjected to flow cytometric analysis.

### Detection of ROS in culture supernatant

The amount of reactive oxygen species present in the supernatants of the T cell cultures was assessed by measuring the oxidation of DCFH-DA in a Wallac Victor 1420 multilabel counter (Perkin Elmer life sciences, C) at wavelength 485/535 nm. The instrument was calibrated with known concentrations of 2',7'-dichlorofluorescein. The cell-free supernatant from the indicated T cell cultures were collected. An equal volume (200 μl) of culture supernatant was taken and DCFH-DA was added at a concentration of 10 μg/ml in a 96 well flat bottom plate (Costar). The plate was incubated at 37°C overnight and fluorescence measured.

### HIV infection in human CD4^+^CD25^- ^T cells

HIV-1_NL4-3 _virus was prepared by transfection of HEK293 T cells as previously described [37]. The virus titer (TCID50 = 10^4.66^/ml) was measured using a Rev-dependent GFP-indicator cell line, Rev-CEM (Ref: Wu and Marsh, Current HIV Research, 2007). 1 × 10^6 ^freshly isolated human CD4^+^CD25^- ^T cells were infected with 150 μl of virus for 2 hours at 37°C, then washed with medium three times to remove free viral particles. Infected cells were seeded onto an anti-human CD3 antibody (5 μg/ml)-coated 24-well plate and cultured with soluble anti-human CD28 antibody (1 μg/ml) for 5 days. A fraction of infected cells was simultaneously cultured with TGF-β (2 ng/ml) or anti-TGF-β antibody (20 μg/ml) on the plate, respectively. Supernatant from infected or uninfected control cells was collected on day 1, 3, and 5. Viral replication was measured by HIV p24 ELISA as recommended by the manufacturer (Beckman Coulter, Fullerton). On days 3 and 5 post-infection, Foxp3/CD25 staining and analysis was performed by flow cytometry as described above.

### Statistical analysis

Student's t tests were used for the significance of data comparison.

## Competing interests

The author(s) declare that they have no competing interests.
